# Novel small ^99m^Tc-labeled affibody molecular probe for PD-L1 receptor imaging

**DOI:** 10.3389/fonc.2022.1017737

**Published:** 2022-10-26

**Authors:** Zhigang Liang, Xianwen Hu, Hongyu Hu, Pan Wang, Jiong Cai

**Affiliations:** Department of Nuclear Medicine, Affiliated Hospital of Zunyi Medical University, Zunyi, Guizhou, China

**Keywords:** programmed death receptor-ligand 1, affibody, SPECT imaging, colon cancer, ^99m^Tc

## Abstract

**Objective:**

The *in vivo* imaging of programmed death ligand 1 (PD-L1) can monitor changes in PD-L1 expression and guide programmed death 1 (PD-1) or PD-L1-targeted immune checkpoint therapy. A ^99m^Tc-labeled affibody molecular probe targeting the PD-L1 receptor was prepared and evaluated its tracing effect in PD-L1-overexpressing colon cancer.

**Methods:**

The PD-L1 affibody was prepared by genetic recombineering. The ^99m^Tc labeling of the affibody was achieved by sodium glucoheptonate and an SnCl_2_ labeling system. The labeling rate, radiochemical purity, and stability *in vitro* were determined by instant thin-layer chromatography; MC38-B7H1 (PD-L1-positive) and MC38 (PD-L1-negative) colon cancer cells were used to evaluate its affinity to PD-L1 by cell-binding experiments. The biodistribution of the ^99m^Tc-labeled affibody molecular probe was then determined in C57BL/6J mice bearing MC38-B7H1 tumors, and tumor targeting was assessed in C57BL/6J mice with MC38-B7H1, MC38 double xenografts.

**Result:**

The nondecayed corrected yield of the ^99m^Tc-PD-L1 affibody molecular probe was 95.95% ± 1.26%, and showed good stability both in phosphate-buffered saline (PBS) and fetal bovine serum within 6 h. The affinity of the ^99m^Tc-PD-L1 affibody molecular probe for cell-binding assays was 10.02 nmol/L. Single photon emission–computed tomography imaging showed a rapid uptake of the tracer in PD-L1-positive tumors and very little tracer retention in PD-L1-negative control tumors. The tracer was significantly retained in the kidneys and bladder, suggesting that it is mainly excreted through the urinary system. Heart, liver, lung, and muscle tissue showed no significant radioactive retention. The biodistribution *in vitro* also showed significant renal retention, a small amount of uptake in the thyroid and gastrointestinal tract, and rapid blood clearance, and the tumor-to-blood radioactivity uptake ratio peaked 120 min after drug injection.

**Conclusion:**

The ^99m^Tc-PD-L1 affibody molecular probe that we prepared can effectively target to PD-L1-positive tumors imaging *in vivo*, and clear in blood quickly, with no obvious toxic side effects, which is expected to become a new type of tracer for detecting PD-L1 expression in tumors.

## Introduction

A blockade targeting PD-1/programmed death ligand 1 (PD-L1) is one of the most promising cancer treatments in cancer therapy, but not all cases respond to these drugs (1–3). The molecular imaging of the immune checkpoint receptor PD-1 and its ligand PD-L1 is receiving increasing attention as a strategy to guide and monitor PD-1/PD-L1-targeted immune checkpoint therapy (4). Compared to the long imaging times of radionuclide-labeled monoclonal antibody (mAb) tracers due to slow blood clearance, low-molecular-weight ligands, such as adherends, nanobodies, or peptides, tend to show faster blood clearance and can be labeled with radioisotopes with shorter half-life, allowing same-day imaging (5). Affibody probes targeting the human epidermal growth factor receptor 2 (HER2) and epidermal growth factor receptor (EGFR) have demonstrated rapid blood clearance and good imaging *in vivo* targeting properties in clinical and preclinical studies (6–12). González Trotter et al. (13) reported the first affibody ligand ^18^F-AIF-NOTA-Z_PD-L1_1_ for detecting PD-L1 expression, and PET imaging showed a good targeting of LOX tumors with high PD-L1 expression.

^99m^Tc is produced by a molybdenum technetium generator, has a suitable gamma ray energy (140.5 KeV) with a short physical half-life (approximately 6 h), and is the most commonly used and ideal radionuclide tracer for single-photon emission computed tomography (SPECT) imaging. In the present study, we developed a new small ^99m^Tc-labeled PD-L1 affibody molecular probe (^99m^Tc-PDA) for SPECT imaging and evaluated its binding properties *in vitro* and *in vivo*, biodistribution, and targeting properties in PD-L1 receptor-positive tumor models. The results demonstrated that the molecular probe has rapid blood clearance and high *in vivo* targeting specificity in PD-L1-overexpressing xenograft tumors.

## Materials and methods

### Materials

Sodium glucoheptonate dihydrate was obtained from TCI (Shanghai, China), dithiothreitol (DTT) from Beijing Xinjingke Biotechnology Co., Ltd. (Beijing, China), tin (II)-chloride dehydrate (SnCl2) and hydroxyethyl piperazine ethanesulfonic acid (HEPES) were purchased from J&K Scientific (Beijing, China), and Ni Sepharose 6FF and Q-Sepharose FF from Beijing Solarbio Science & Technology Co., Ltd. (Beijing, China). MC38 and MC38-B7H1 mouse colon cancer cells were acquired from BMCR (Beijing, China), the instant thin-layer chromatography silica gel (iTLC-SG) chromatography paper was purchased from Agilent Technologies (Palo Alto, CA, USA) and NAP-5 size exclusion columns from GE Healthcare (Uppsala, Sweden), C57BL/6J Mice were purchased from Hunan SJA Laboratory Animal Co (Changsha, China). The GC-2010 gamma radiation counter was purchased from USTC ZONKIA (Hefei, China), the Infinia V Hawkeye 4 SPECT/CT imaging system was purchased from GE Healthcare (Chicago, IL, USA), and ^99m^Tc was obtained as pertechnetate from an ^99^Mo/^99m^Tc Generator (HTA, Beijing, China) eluted with sterile 0.9% sodium chloride.

### Preparation of programmed death ligand 1 affibody

*Escherichia* coli BL21 cells were transformed with plasmids pET26b (+) containing a gene fragment encoding PD-L1 targeting affibody (shorted for PDA) with a histidine–glutamate–histidine–glutamate–histidine–glutamate (HEHEHE)-tag at the amino terminus and a glycine–glycine–glycine–cysteine (GGGC) chelator at the carboxyl terminus, with the amino acid sequence of MAHEHEHEAEAKYAKERNKAAYEILYLPNLTNAQKWAFIWKLDDDPSQSSELLSEAKKLNDSQAPKGGGSGGGC. Cells were cultivated in an LB medium containing 50 μg/ml kanamycin at 37°C, and protein expression was induced by 1 mmol/L isopropyl-β-D-thiogalactoside (IPTG). After harvesting, cells were disrupted by sonication followed by centrifugation to remove cell debris, and the clarified cell lysate was heat-treated at 60°C for 10 min to precipitate a portion of endogenous *E. coli* proteins. The heat-treated cell lysate was then centrifuged and filtered through a 0.22 μm filter. Affibodies were recovered by immobilized metal affinity chromatography (IMAC, Ni Sepharose 6FF) and further purified by anion exchange (Q-Sepharose FF).

The purified affibodies were identified by sodium dodecyl sulfate–polyacrylamide gel (SDS-PAGE) and matrix-assisted laser desorption ionization–tandem time-of-flight mass spectrometry (Maldi-TOF/TOF).

### Radiolabeling and *in vitro* stability of ^99m^Tc-PDA

The purified PDA was added with DTT at a final concentration of 30 mmol/L, reduced at 37°C for 2 h to destroy the spontaneously formed intermolecular disulfide bonds between cysteines, desalted on a NAP-5 column, and stored at 4°C.

A sterile vial was added with 200 μl of an argon-degassed labeling buffer (10 mM HEPES, 20 mM sodium glucoheptonate, pH 6.6), 100 μl of a reducing affibody (approximately 80 μg), 100 μl of ^99m^TcO_4_ (typically, 37 MBq), and 0.5 µl of a freshly prepared SnCl_2_ solution (0.4 mg/ml, in 0.5 mmol/L hydrochloric acid). The reaction solution was incubated at 90°C for 10 min and then cooled at room temperature for 15 min, followed by filtered through a 0.22-micron filter and diluting with PBS.

TLC was performed as described in the reference (14). A small aliquot of samples (~ 0.5 μl) was taken for the TLC analysis of the labeling yield using iTLC-SG with the PBS mobile phase and of reduced hydrolyzed technetium colloid levels using iTLC-SG with a 10:6:3 ratio (pyridine:acetic acid:water) as the mobile phase.

To test the labeling stability *in vitro*, three 100-μl aliquots of ^99m^Tc-PDA were mixed with 900 μl of either PBS or fetal bovine serum. Each 0.5 μl sample was taken for TLC analysis, respectively, after incubation for 0.5, 1.0, 2.0, 4.0, and 6.0 h at 37°C.

### Cell lines

Colon cancer MC38 cells and human PD-L1 gene-transfected MC38 cells (MC38-B7H1) were cultured in Roswell Park Memorial Institute (RPMI) 1640 medium containing 10% fetal bovine serum and 1% penicillin–streptomycin at 37°C in a 5% CO_2_ incubator. While they reached confluence, cells were passaged using a trypsin containing 0.25% ethylene diamine tetraacetic acid (Trypsin-EDTA) solution.

### Binding specificity and cellular uptake

The binding affinity and specificity of ^99m^Tc-PDA to human PD-L1 were determined in MC38-B7H1 cells using MC38 cells as a negative control. MC38-B7H1 and MC38 cells were seeded into 24-well plates at a density of 2 × 10^5^ cells/well and cultured overnight at 37°C. At confluency, cells were treated with increasing concentrations (0.02, 0.10, 0.52, 2.62, 13.11, 65.53, 327.63, and 1638.14 nM, three wells for each concentration) of ^99m^Tc-PDA. After incubation at 4°C for 2 h, the medium was discarded, the cells were washed three times with ice-cold PBS and then lysed with 0.1 M NaOH, and cell lysates were collected. The radioactivity of ^99m^Tc-PDA that was bound to the cells was measured by a gamma counter. The dissociation constant (K_D_)values were calculated by nonlinear fitting (one-site total and nonspecific binding) using GraphPad Prism 6 software.

The specific binding of ^99m^Tc-PDA to human PD-L1 was also confirmed in a competitive binding experiment using MC38-B7H1 cells with high PD-L1 expression. A set of six dishes containing a cell monolayer (10^6^ cells/dish) were incubated with 1.5 nM ^99m^Tc-PDA for 1 h; cells in three dishes were added a 100-fold excess of an unlabeled affibody 5 min before the addition of ^99m^Tc-PDA. After incubation, the medium was collected and the cells were washed three times with ice-cold PBS followed by treatment with 0.1 M NaOH to collect cellular bound radioactivity, and the percentage of cell-bound radioactivity was calculated.

Cellular uptake and the internalization of ^99m^Tc-PDA were studied using PD-L1-expressing colon cancer MC38-B7H1 cells. Briefly, cells (10^6^ cells/dish) were incubated with labeled conjugates (1.5 nM) at 37°C. At predetermined time points (1, 2, 4, 8, 12, and 24 h after incubation started), the supernatant from a set of three dishes was collected and the cells were washed twice with ice‐cold PBS; the combined fractions represent the unbound radioligand. The cells then were treated with a buffer containing 4 M urea and 0.2 M glycine, pH 2.5 for 5 min on ice, and membrane-bound radio conjugates were collected. The internalized affibodies were collected after the cells were lysed with 1 M NaOH finally. The percentage of membrane-bound and internalized radioactivity was calculated for each time point.

### Animal models

All animal experiments were approved by the principles of the Ethics Committee of Affiliated Hospital of Zunyi Medical University (grant number, KLLYA-2021-019). Female C57BL/6J mice, 7 weeks old, were housed in ventilated filter-topped cages with free access to a standard diet and water. Approximately 10^6^ of MC38-B7H1 cells (in 100 μl PBS) were implanted subcutaneously in the right armpit. The mice were used for biodistribution studies approximately 2 weeks after injection, when the tumor reached a volume of approximately 1 cm^3^.

Dual-tumor xenograft mice were generated by the implantation of 10^6^ of both MC38-B7H1 cells and MC38 cells in each armpit, which allows ^99m^Tc-PDA to assess radioactive uptake in tumors with low and high PD-L1 receptor expression in the same animal. SPECT imaging is performed when the tumor volume reaches approximately 1 cm^3^, The expression of PD-L1 in these tumors was determined by immunohistochemistry with a rabbit anti-human PD-L1 monoclonal antibody (ZR3).

### *Ex vivo* biodistribution studies

MC38-B7H1 tumor-bearing mice were injected with approximately 1.85 MBq of ^99m^Tc-PDA *via* the lateral tail vein, which corresponds to approximately 4 μg of peptide. Mice were sacrificed immediately (approximately 10 s), 30, 60, 120, 180, and 360 min after injection. The tumor, heart, liver, spleen, lung, kidney, brain, thyroid, muscle, bone (femur), stomach, duodenum, and eyeball blood were collected and weighed, and radioactivity was measured using a gamma counter. Biodistribution measurements were expressed as percent injected dose rate per gram tissue (%ID/g).

### SPECT imaging

Dual-tumor xenograft mice were anesthetized with isoflurane (4%–5% induction, 1%–3% maintenance), and approximately 3.7 MBq of ^99m^Tc-PDA (approximately 8 μg PDA) was injected *via* the lateral tail vein. SPECT imaging was performed at 30, 60, and 120 min after injection, and 200 K counts were collected. Mice in the blocking group were injected with 400 μg of unlabeled affibodies 5 min before the tail vein injection of ^99m^Tc-PDA, and SPECT imaging was performed 60 min later.

### Statistical analysis

Statistical analysis was performed using Statistical Product and Service Solutions (SPSS) software version 25.0, and the mean ± standard deviation ( $\bar{\text{x}}$ ± s) was used to represent the measurement data conforming to the normal distribution. The comparison between two groups was performed by an independent sample *t*-test, and the comparison among multiple groups was performed by one-way analysis of variance (one-way ANOVA). If *P*<0.05, the pairwise mean difference test (i.e., least significant difference method) was further used for pairwise comparison when the variances were equal, Tamhane’s T2 test or Dunnett’s T3 test was used when variances were unequal, and *P*<0.05 was considered statistically significant for differences.

## Results

### Production, purification, and characterization of PDA

**Figure 1:**
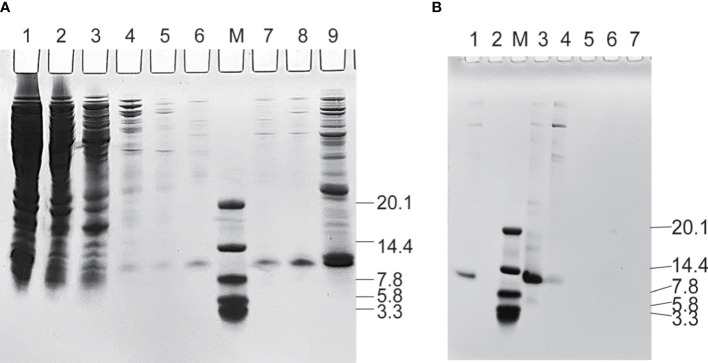
Sodium dodecyl sulfate–polyacrylamide gel analysis during the purification of PDA. **(A)** Purification of affibodies by immobilized metal affinity chromatography. Lane M, the protein molecular marker (kDa), Lanes 1~9 are cell lysate after heat treatment, unbound protein, 5, 10, 20, 30, 40, 60, and 300 mM imidazole-eluted product, respectively. **(B)** Purification of affibodies by anion exchange. Lane M, the protein molecular marker (kDa); Lane 1~7 sequentially are sample eluted with 60 mM imidazole and 50, 200, 500, 800, 1,200, and 4,000 mM NaCl-eluted products.

**Figure 2:**
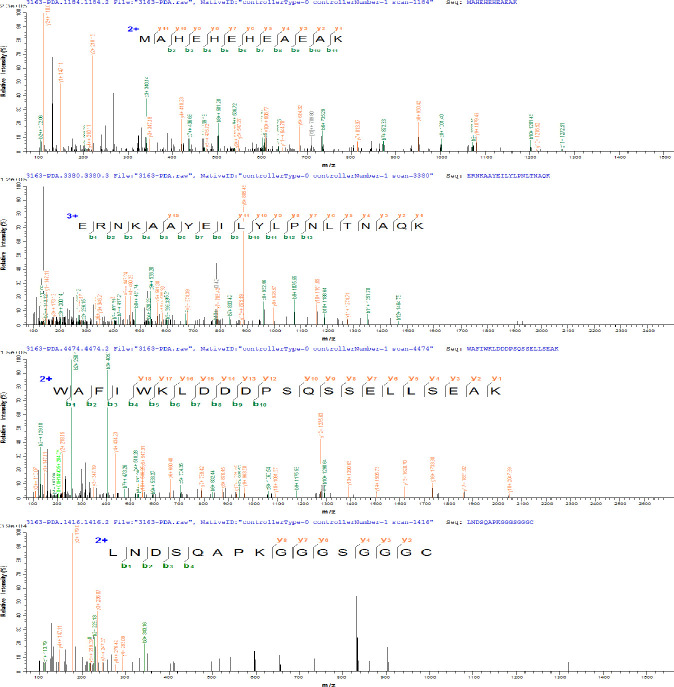
Amino acid sequence of PDA determined by matrix-assisted laser desorption ionization–tandem time-of-flight mass spectrometry (Maldi-TOF/TOF).

PDA was expressed in *E*. *coli* and recovered by IMAC. The imidazole gradient was further divided, and it was found that elution with 60 mM imidazole yields a purity more than 90% (as shown in **Figure 1A**), which was further purified by anion exchange and was eluted with 200 mM NaCl to a purity of >95% (as shown in **Figure 1B**). The purified affibodies were confirmed by Maldi-TOF/TOF (as shown in **Figure 2**), showing that the amino acid coverage was 95%, and the molecular mass was 8,227 Da.

### Radiolabeling and *in vitro* stability of ^99m^Tc-PDA

**Figure 3:**
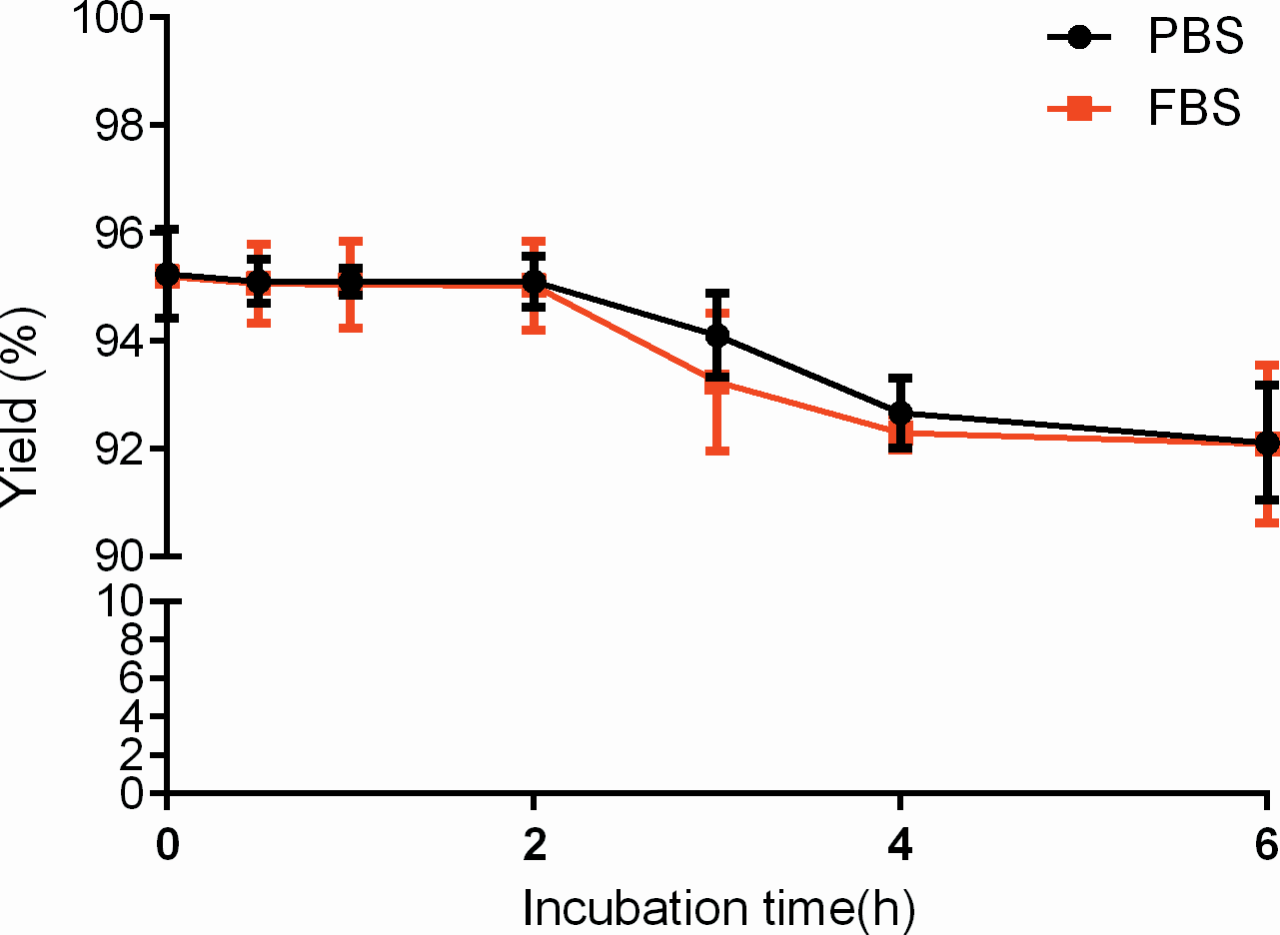
Stability of ^99m^Tc-labeled PD-L1 affibody molecular probe (^99m^Tc-PDA) in phosphate-buffered saline and serum.

^99m^Tc-PDA was obtained with a high labeling yield (95.95% ± 1.26%); reduced hydrolyzed technetium colloid (3.21% ± 0.37%) (n=10) could be used for biological experiments without additional purification. Bacterial endotoxin assay results show less than 1 EU/ml. TLC analysis showed that ^99m^Tc-PDA had good stability, and the radiochemical purity was decreased slightly after 4~6 h of incubation in PBS and serum at 37°C (*p<*0.05) and still greater than 90% (as shown in **Figure 3**).

### Binding specificity and cellular uptake

**Figure 4:**
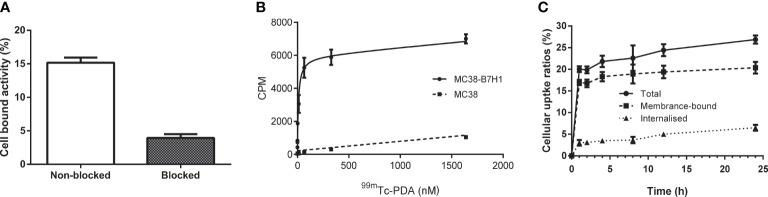
Binding specificity and cellular uptake. **(A)** Binding specificity of ^99m^Tc-PDA to PD-L1 in competitive binding assays. **(B)** Affinity analysis of ^99m^Tc-PDA to PD-L1-expressing MC38-B7H1 cells. **(C)** Uptake and internalization of ^99m^Tc-PDA at 37°C by MC38-B7H1 cells.

Radioactivity was significantly higher in MC38-B7H1 than in MC38 cells at all concentration points (*P*<0.01), and competitive binding assays showed that excess unlabeled affibodies obviously reduced the binding of ^99m^Tc-PDA to MC38-B7H1 cells,which suggests that the binding of ^99m^Tc-PDA to living PD-L1-expressing cells was receptor mediated (as shown in **Figure 4A**). ^99m^Tc-PDA had a high affinity to MC38-B7H1 cells with a K_D_ value of approximately 10.02 nM (as shown in **Figure 4B**).

Binding of ^99m^Tc-PDA to MC38-B7H1 cells increased rapidly during the first 1 h of culture; only a minor increase was observed after this time. The internalization of ^99m^Tc-PDA by MC38-B7H1 cells increased with time; about 24.25% ± 2.99% of the total cell-associated radioactivity was internalized after 24 h of incubation (as shown in **Figure 4C**).

### Identification of MC38/MC38-B7H1 xenograft model

**Figure 5:**
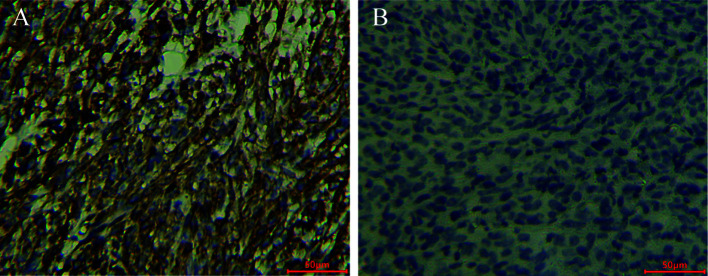
ZR3 immunohistochemical staining of MC38-B7H1 and MC38 xenograft tumors Scale bar: 50 μm; **(A)** Membranous staining was observed in MC38-B7H1 samples. **(B)** There was no signal in MC38 tumors.

Immunohistochemistry (IHC) confirmed the strong positive expression of PD-L1 in MC38-B7H1 xenograft tumors and the negative expression of PD-L1 in MC38 tumors (as shown in **Figure 5**).

### Biodistribution in MC38-B7H1 xenograft-bearing mice

The data concerning the biodistribution of ^99m^Tc-PDA in MC38-B7H1 xenograft mice are presented in **Table 1**. The molecular probe ^99m^Tc-PDA showed rapid clearance from the blood (14.13 ± 1.59%ID/g at 10 s after injection, whereas 0.50 ± 0.12%ID/g at 60 min after injection). The heart, spleen, and lung also showed the highest ^99m^Tc-PDA uptake immediately after injection and then gradually decreased along with time. Renal retention was obvious; the uptake was the highest at 30 min after injection (%ID/g=87.53 ± 15.09) and gradually decreased with time and decreased to at 360 min (%ID/g=5.63 ± 1.61).

**Table 1:** Biodistribution of ^99m^Tc-labeled PD-L1 affibody molecular probe in MC38-B7H1 xenograft-bearing mice (%ID/g, $\bar{\text{x}}$ ± s).

Organ or tissue	10 s (n = 3)	30 min (n = 7)	60 min (n = 6)	120 min (n = 7)	180 min (n= 8 )	360 min (n = 3)
Blood	14.13 ± 1.59	2.11 ± 0.58	1.04 ± 0.25	0.50 ± 0.12	0.33 ± 0.08	0.11 ± 0.003
Heart	3.01 ± 0.13	1.98 ± 0.36	0.85 ± 0.12	0.57 ± 0.05	0.30 ± 0.07	0.09 ± 0.01
Liver	2.71 ± 0.46	3.50 ± 0.53	2.51 ± 0.24	2.00 ± 0.31	2.03 ± 0.32	2.01 ± 0.04
Spleen	2.32 ± 0.44	1.70 ± 0.35	0.81 ± 0.14	0.77 ± 0.17	0.71 ± 0.11	0.58 ± 0.02
Lung	5.55 ± 1.47	4.10 ± 0.68	2.03 ± 0.27	1.66 ± 0.41	0.87 ± 0.26	0.43 ± 0.08
Kidney	8.37 ± 1.54	87.53 ± 15.09	35.27 ± 3.08	17.86 ± 2.13	12.96 ± 1.94	5.6 3± 1.61
Brain	0.55 ± 0.07	0.14 ± 0.03	0.11 ± 0.03	0.11 ± 0.02	0.04 ± 0.01	0.02 ± 0.005
Thyroid	2.45 ± 0.28	3.63 ± 0.56	3.69 ± 0.26	4.50 ± 0.89	2.75 ± 0.74	0.41 ± 0.09
Gastrointestinal	1.59 ± 0.09	4.19 ± 0.94	4.24 ± 1.03	4.82 ± 1.09	2.71 ± 0.73	0.17 ± 0.04
Tumor	1.03 ± 0.15	12.06 ± 1.61	14.97 ± 3.47	15.50 ± 3.68	8.77 ± 2.70	0.98 ± 0.07
Muscle	0.57 ± 0.09	1.34 ± 0.22	0.77 ± 0.15	0.37 ± 0.07	0.29 ± 0.07	0.09 ± 0.02
Bone	1.80 ± 0.28	2.09 ± 0.47	1.62 ± 0.31	0.38 ± 0.07	0.88 ± 0.24	0.18 ± 0.04

**Figure 6:**
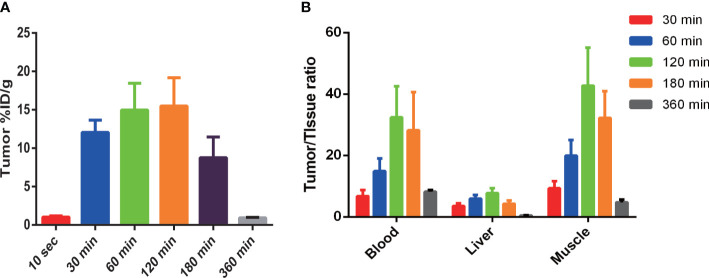
^99m^Tc-PDA uptake in MC38-B7H1 tumors **(A)** and comparison of ^99m^Tc-PDA in tumor to tissue **(B)**. **(A)** Tumor uptake was significantly higher at all time points after 30 min than 10 s post-^99m^Tc-PDA injection (*P*< 0.01). **(B)** Significant difference in tumor-to-blood ratios were observed at 30 min vs. 120 min and 60 min vs. 120 min after injection (*P* < 0.01); the tumor-to-liver ratios were significantly higher at 120 min compared to 30 and 180 min (*P* < 0.01), and the tumor-to-muscle ratios were significantly higher at 120 min compared to 30 and 60 min.

Tumors exhibited rapid uptake, with a significant increase in tracer tumor uptake after 30 min followed by a slow increase and a gradual decrease after peaking at 120 min. The %ID/g ratio of tumors compared with blood, the liver, and muscle reached a peak at 120 min after injection, and the ratios were (32.40 ± 10.18), (7.79 ± 1.54), and (42.72 ± 12.44), respectively (as shown in **Figure 6**).

### SPECT imaging of dual-flank MC38-B7H1/MC38 xenograft-bearing mice

**Figure 7:**
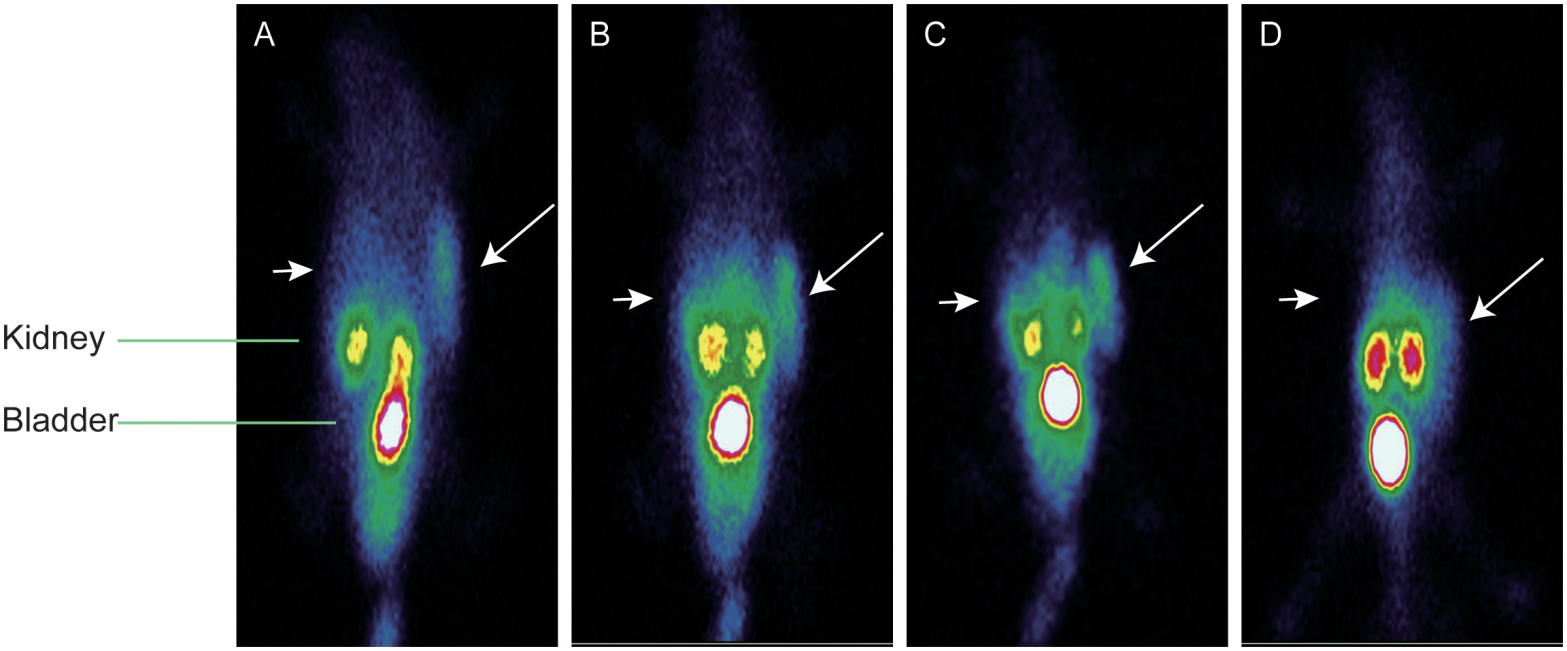
Representative single photon emission–computed tomography maximum-intensity projection images of ^99m^Tc-PDA in dual-flank MC38-B7H1/MC38 xenograft-bearing mie; long arrows indicate MC38-B7H1 tumors, and short arrows indicate MC38 tumors. **(A)** Images at 30 min. **(B)** Images at 60 min. **(C)** Images at 120 min. **(D)** Blocking images at 60 min.

The SPECT imaging of tumor-bearing mice used an imaging system for the human body; the spatial resolution of the images was therefore limited. However, the radiosonde accumulation in the MC38-B7H1 tumor was clearly observed 30 min after injection, while the MC38 tumor was never visible. The highest radioactive accumulation was demonstrated in bilateral kidneys and the bladder, indicating that ^99m^Tc-PDA was mainly excreted through the urinary system, as previously observed in the biodistribution studies. The thyroid and stomach were not visualized, conjecturing that ^99m^Tc-PDA had good stability *in vivo* within 120 min. A blockade with 400 μg of PDA caused a significant reduction of ^99m^Tc-PDA uptake in MC38-B7H1 tumors (as shown in **Figure 7**). We manually demarcated the tumor and lower extremity (representing the radioactivity of the muscle) and obtained the mean of the radioactivity counts per unit volume, and the ratio of radioactivity counts of the tumor to muscle was 17.84 ± 2.80 at 120 min after injection, which is lower than in biodistribution.

## Discussion

High PD-L1 expression is associated with a poor prognosis in various malignant tumors (15, 16); blockades targeting the PD-1/PD-L1 pathway have shown controllable safety and durable remissions in lung cancer, melanoma, bladder cancer, and other tumors (17–28). However, 30%–60% of patients do not respond to the PD-1/PD-L1 blockade (2). In addition, considering the immune-related adverse events of the drugs (29, 30) and the high cost of treatment, it is of great significance to screen patients who can benefit from these drugs before treatment.

The efficacy of PD-1/PD-L1 blockade therapy differs significantly between PD-L1-positive and PD-L1-negative patients (31). The expression of PD-L1 is currently one of the most important predictive biomarkers, and the detection of PD-L1 expression in the tumor environment by IHC is the most widely used patient screening method for immunotherapy at present. However, IHC results are adversely affected by biopsy sampling errors and the consistent ratio of PD-L1 expression in primary tumors and metastatic lesions (32, 33), and static biopsies cannot dynamically monitor changes in PD-L1 expression during treatment. Molecular imaging using radiolabeled enables a noninvasive, comprehensive, and dynamic assessment of PD-L1 expression *in vivo*.

Currently, commonly used molecular imaging probes targeting PD-L1 include antibodies, small peptides, or proteins that specifically bind to the receptor. Monoclonal antibodies have high affinity and specificity; however, due to their large molecular weight and weak tissue penetration, which has long circulation retention time, the needed labeling with isotopes with a longer half-life and high-contrast imaging can often only be performed after multiple days and with a risk of false-positive results due to the remaining blood pool activity. Furthermore, the use of long-lived isotopes risks increasing the exposure of subjects to radiation (34–36). Proteins that bind specifically to receptors such as affibodies is an artificial non-immunoglobulin molecule derived from the B domain of staphylococcal A protein with a molecular weight of approximately 6.5 kDa, consisting of 58 amino acid residues and lacking cysteine (37). The introduction of cysteine can provide a binding site for the specific site binding of thiol-reactive radionuclides or chelates. In our present study, the GGGC sequence was introduced into the carboxyl terminus of the PD-L1 affibody, and the thiol group of cysteine together with the amide nitrogen of the adjacent amino acid formed an N_3_S chelate structure, thereby realizing the stable labeling of ^99m^Tc (14, 38, 39). Moreover, a hydrophilic HEHEHE-tag was introduced at the N-terminus of the PD-L1 affibody, on one hand, to facilitate the recovery of protein by IMAC; on the other hand, the hydrophobicity of the HEHEHE-tag reduces the hepatic retention of the tracer (40–42).

^99m^Tc-PDA exhibits very rapid blood clearance, which is associated with its rapid excretion through the urinary system. The uptake of the molecule probe in the heart and lung was highest immediately after injection, which was related to the abundant blood supply of these organs. Both biodistribution studies and *in vivo* imaging showed low uptake in the spleen, indicating that PDA does not cross-react with murine PD-L1. In addition, increased radioactive uptake in the thyroid and gastrointestinal tract was observed within 30–120 min postinjection; this was presumably associated with a small amount of unconjugated ^99m^TcO in the labeled compound. ^99m^TcO can be absorbed by a normal thyroid and gastric mucosa and secreted by the gastric mucosa and then enter the intestinal tract. Sustained decrease was observed over the subsequent time, indicating that ^99m^Tc-PDA was stable *in vivo*.

^99m^Tc-PDA has a high affinity for the PD-L1 receptor, and the K_D_ value reaches the nM level, similar to the previously reported PD-L1 affibody molecular probes (13, 43). Compared with ^18^F and ^68^Ga labeled affibodies, ^99m^Tc-PDA had lower renal retention, which may be explained by differences in radioisotopes and the use of bifunctional chelators.

We had tested the detection ability of ^99m^Tc-PDA for PD-L1 expression at different times after injection, and the results showed that ^99m^Tc-PDA could quickly penetrate into the tumor tissue after injection; high-contrast tumor imaging could be obtained within 30 min. Combined with the characteristics of biodistribution, it is speculated that the optimal imaging time is 1~2 h after injection, comparable to the imaging time of peptide- or nanobody-based imaging agents (44–46), which is associated with the rapid blood clearance and tumor penetration of small-molecular-weight ligands. Compared with mAb-based tracers (35, 47), imaging time was significantly shortened, and liver retention was also reduced due to the different metabolic pathways between affibodies and antibodies.

There were still some shortcomings in this study such as unsatisfactory image quality due to SPECT imaging using an imaging system for the human body and a higher uptake of the tracer in the kidneys, which would limit the injected activity, resulting in reduced sensitivity to low-expressing lesions. The alteration of the affibody structure is needed to reduce renal uptake in future research. Furthermore, the uptake of tracers in the thyroid and gastrointestinal tract was relatively high although significantly lower than in tumors, optimizing the formulation of the labeling system, so improving the labeling rate is an effective method to solve such problems.

In a nutshell, our developed molecular probe ^99m^Tc-PDA showed rapid blood clearance and good targeting *in vivo* and is expected to be a candidate drug for the SPECT/CT imaging of PD-L1 expression in cancer patients. Further clinical studies are needed to clarify its metabolic characteristics and imaging potential in humans.

## Data availability statement

The original contributions presented in the study are included in the article/supplementary material. Further inquiries can be directed to the corresponding authors.

## Ethics statement

The animal study was reviewed and approved by Institutional Animal Ethics and Use Committee of Zunyi Medical University, Zunyi Medical University.

## Author contributions

JC and ZL designed the experiments; ZL and XH performed the experiments and wrote the original manuscript; HH analyzed the data; JC and PW revised the manuscript. All authors contributed to the article and approved the submitted version.

## Funding

This study was funded by ZunShiKeHe HZ2014214, Zunyi Medical College Research Start Fund 2018ZYFY03.

## Conflict of interest

The authors declare that the research was conducted in the absence of any commercial or financial relationships that could be construed as a potential conflict of interest.

## Publisher’s note

All claims expressed in this article are solely those of the authors and do not necessarily represent those of their affiliated organizations, or those of the publisher, the editors and the reviewers. Any product that may be evaluated in this article, or claim that may be made by its manufacturer, is not guaranteed or endorsed by the publisher.
